# Effect of Educational Intervention by Application of PRECEDE-PROCEED Model on Lifestyle Change in Hypertensive Patients

**DOI:** 10.1155/2024/5523473

**Published:** 2024-10-24

**Authors:** Alireza Ghannadi, Fatemeh Mohammadkhah, Pooyan Afzali Harsini, Afsaneh Ghasemi, Amirhossein Kamyab, Ali Khani Jeihooni

**Affiliations:** ^1^Departement of Public Health, School of Health, Fasa University of Medical Sciences, Fasa, Iran; ^2^Nursing Care Research Center, Health Research Institute, Babol University of Medical Sciences, Babol, Iran; ^3^Departement of Public Health, School of Health, Kermanshah University of Medical Sciences, Kermanshah, Iran; ^4^Faculty of Medicine, Fasa University of Medical Sciences, Fasa, Iran; ^5^Departement of Public Health, School of Health, Shiraz University of Medical Sciences, Shiraz, Iran

**Keywords:** education, hypertensive patients, lifestyle, PRECEDE-PROCEED model

## Abstract

**Background:** One of the most important causes of cardiovascular disease is hypertension. Lifestyle modification has been emphasized in preventing and controlling blood pressure. This research aimed to determine the effect of educational intervention by application of PRECEDE-PROCEED model on lifestyle change in hypertensive patients in the villages of Fasa City, Fars Province, Iran.

**Methods:** This research is a quasi-experimental study that was conducted on 300 hypertensive patients in 2020–2021. Data gathering tools were a demographic information questionnaire, a questionnaire based on the PRECEDE-PROCEED model, and a lifestyle questionnaire (LSQ). The educational intervention consisted of ten sessions lasting 45 or 50 min. Before and 6 months after the educational intervention, the two groups each completed a questionnaire. The systolic and diastolic blood pressure (DBP) levels, as well as physical exercise and health variables, nutrition and weight control, mental health, and spiritual health were measured before and 6 months after the educational intervention.

**Results:** The results showed that there was no significant difference between the two groups before in cues of PRECEDE-PROCEED model; however, the experimental group had a significant increase in cues of PRECEDE-PROCEED model 6 months after the intervention. The results showed that the experimental group had a significant increase 6 months after the intervention in terms of physical exercise and health variables, nutrition and weight control, mental health, and spiritual health. The mean blood pressure (both diastolic and systolic) in the experimental group was significantly reduced after the intervention.

**Conclusions:** The study's results showed the effectiveness of an educational intervention based on the PRECEDE-PROCEED model on lifestyle change in hypertensive patients. It also highlights the need to pay further attention to the education aimed at controlling hypertension through a healthy lifestyle and correct behavioral habits.

## 1. Background

Hypertension has become a significant worldwide health issue for primary healthcare systems [1]. Hypertension is one of the leading causes of cardiovascular disease, early death, and overall mortality worldwide [2].

The prevalence hypertension is 1.39 billion adults in worldwide, or 31.1% of the adult population. Hypertension is responsible for roughly 17% of all deaths [3, 4]. Hypertension is becoming more common. Between 1990 and 2019, 626 million women and 652 million men had hypertension, compared to 331 million women and 317 million men in 1990 [5]. In 2015, for both sexes Australia, Canada, Peru, Singapore, South Korea, the United Kingdom, and the United States of America were the nations with the lowest prevalence of hypertension [6]. Adults with hypertension grew from 1.8 million in 1990 to 13.6 million in 2016 in Iran [7]. In 2019, there were 23% more women than men who had hypertension under control [5]. Systolic blood pressure (SBP.) greater than 140 mmHg or diastolic blood pressure (DBP) greater than 90 mmHg is considered elevated blood pressure [6]. Throughout a person's life, dietary, environmental, and behavioral factors can impact blood pressure [6]. Of the 774 persons with hypertension, 84.9% were unaware of it, 63.2% for not brushing their teeth at least twice a day, 35.9% for using tobacco or alcohol, 53.9% for being overweight or obese, 17.1% for not exercising in any way, and 92.3% for failing to ingest at least five F.V.^1^ servings per day. Additionally, in the Middle East region several modifiable factors (including socioeconomic factors and disparities in education, literacy, and urbanization, as well as excessively high rates of smoking, obesity, a sedentary lifestyle, and a poor healthcare system), have been linked to the prevalence of hypertension [8, 9].

In Iran about 23.39 million people suffer from moderate SBP (BP of 120 to 139 mm/Hg) and a further 14.6 million people had severe BP (≥140 mm/Hg). Nearly 40% of these patients, receive BP treatment [10].

Blood pressure control has been related to several characteristics (including sex, age, education, medication burden, comorbidity, and dietary habits such as high salt consumption, alcohol, cigarettes, body mass index, and exercise) [11, 12]. A lifestyle is a pattern of daily conduct that a social group in the community should adhere to following the law and religion [13].

Despite the availability of health education and advice regarding hypertension, its effects and lifestyle modification, many hypertensive patients do not adhere to healthy lifestyle practices [14]. Moreover, one adequately provided health education could lead to change in lifestyle behavior [15]. Controlling hypertension requires adopting appropriate treatments to improve the knowledge and attitude of hypertensive patients and adherence to the prescribed treatment plan [16].

The lifestyle modification is a key component of the antihypertension program, in which education is crucial [17]. Education must be based on accepted ideas and models to provide successful results [18]. One framework and design pattern for identifying health education and promotion needs is the PRECEDE-PROCEED model. This model can change behavior and examine educational programs' possible outcomes. The cues of this model are predisposing factors, reinforcing factors, and enabling factors [19].

Considering the increasing number of patients with high blood pressure, the heavy financial burden of the disease, and WHO's emphasis on the importance of lifestyle modification in blood pressure control, the role of education in lifestyle modification, and the role of theories in the effectiveness and usefulness of education and paying attention to the PRECEDE-PROCEED model as a framework and model for identifying educational needs, creating a process to change behavior and examine the possible results of the educational program, the present study aimed to evaluate the effect of educational intervention by application of the PRECEDE-PROCEED model on lifestyle change in hypertensive patients.

The lifestyle modification is a key component of the antihypertension program, in which education is crucial [16]. Education must be based on accepted ideas and models to provide successful results. One framework and design pattern for identifying health education and promotion needs is the PRECEDE-PROCEED model. The present study aimed to evaluate the effect of educational intervention by application of PRECEDE-PROCEED model on lifestyle change in hypertensive patients.

## 2. Methods

### 2.1. Study Design and Participants

This research was a quasi-experimental study conducted on 300 hypertensive patients in the villages of Fasa City in 2020–2021. The statistical population of the research was all patients with high blood pressure referred to rural health centers of Fasa City, Fars, South of Iran.

### 2.2. Sample Size and Sampling Method

The sample size for this study was determined based on similar studies and calculated using G-Power software [20]. With a confidence level of 95%, a power of 80%, and considering the mean and standard deviation of lifestyle scores (30.5 ± 2.8 in the intervention group and 29.27 ± 3.3 in the control group), the required sample size was estimated to be 150 participants per group. Additionally, an attrition rate of 20% was considered, leading to a total sample size of 300 hypertensive patients.

The sampling method used in this study was a multistage clustering approach. Initially, the study area consisted of eight rural health centers in Fasa City, Fars Province, Iran. These centers provide outpatient services and have comprehensive coverage.

1. Cluster selection: Eight rural health centers were chosen at random for the first phase. Each center was considered a cluster.
2. Randomization: Four out of these eight health centers were randomly selected to form the experimental group, while the remaining four were assigned to the control group.
3. Patient selection: Within each health center, patients were selected randomly to meet the required sample size. Specifically, 37 patients were chosen from each health center, totaling 150 patients for both the experimental and control groups.
4. Randomization process: The randomization was executed using a computer-generated random number table to ensure unbiased selection and allocation of participants into the intervention and control groups. This process ensured that every eligible patient had an equal chance of being included in either group, thus maintaining the study's validity and reliability.

In summary, a total of 300 hypertensive patients were randomly assigned to either the intervention group (*n* = 150) or the control group (*n* = 150), ensuring a balanced distribution across the selected health centers.

### 2.3. Ethical Considerations

Ethical approval was obtained from the Human Research Ethics Committee at the Fasa University of Medical Sciences. All study participants provided written informed consent. Permission was also obtained to digitally record all interview. Informed consent from legally authorized representatives for study participation for illiterate participants. Confidentiality and anonymity were ensured. The ethics committee approved the procedure for verbal consent since the study is observational and respected the code of ethics as stated in the declarations of Helsinki. Also, the present study was approved by (Ethical Code: IR.FUMS.REC.1400.031). Moreover, written consent was obtained from the participants, and their information would be treated as strictly confidential.

### 2.4. Inclusion and Exclusion Criteria

The inclusion criterion was having a health record at a health center, being hypertensive for at least a year, which was diagnosed by a doctor and patients with a diagnosis of secondary hypertension with consent for participants in the study were eligible to be enrolled into the study. The exclusion criterion was absenteeism in more than two sessions of educational classes, unwillingness to cooperate at any time of the study, or migration and pregnant women with high blood pressure.

### 2.5. Data Collection Tools

Data collection tools were based on similar studies [21–24], which included: demographic characteristics, PRECEDE-PROCEED model questionnaire based (A researcher-made questionnaire), and a lifestyle questionnaire (the standard questionnaire of Lilly et al. [25]).

### 2.6. PRECEDE Constructs

The PRECEDE-PROCEED model questionnaire included knowledge, attitude, self-efficacy, enabling, and reinforcing factors.

• Knowledge: This section have 25 questions based on a 2-point scale (one correct response, zero erroneous responses), regarding the knowledge of hypertension, and the scores were between 0 and 25 (e.g., “If I don't eat less fatty foods, I will most likely in the future I will have blood pressure complications (such as heart attack or stroke)”.
• Attitude: This section have 25 questions based on a 5-point Likert scale [the responses ranged from “completely disagree” (scoring zero) to “completely agree” (score four), on a 5-point Likert scale], scores were between 0 and 100. These questions assessed attitudes toward hypertension (e.g., “I feel that the effects of blood pressure can be reduced by consuming low-fat foods”.
• Self-efficacy: This section have 15 questions based on a 5-point Likert scale [completely disagree (scoring zero) to “completely agree” (score four), on a 5-point Likert scale], scores were between 0 and 60. These questions measured self-efficacy of participants in coping with hypertension by following a healthy lifestyle (for example: I can exercise every day).
• Enabling variables: This section have 15 questions based on a 5-point Likert scale [completely disagree (scoring zero) to “completely agree” (score four), on a 5-point Likert scale], scores were between 0 and 60 (e.g., “I have access to sufficient educational materials in the field of lifestyle modification related to hypertension patients”).
• Reinforcing factors: This section have six questions on the support of family members (parents, siblings, etc.), other relatives, friends, doctors, and health center staff. The answers were on a 5-point Likert scale ranging from “completely disagree” (score zero) to “completely agree” (score four); scores were 0 and 24 (e.g., “My family members encourage me to exercise regularly”).

### 2.7. Validity and Reliability of the Questionnaire

Face validity was determined in this study using both qualitative and quantitative methodologies. At the qualitative stage, 40 patients with similar demographic, socioeconomic, and social backgrounds were interviewed in person to evaluate the difficulty, relevancy, and ambiguity of each item. To decrease and eliminate unnecessary items and assess each item's importance, the quantitative approach of item impact was applied in the following stage.

Both quantitative and qualitative methods assessed content validity. In the qualitative phase a panel (of 10 experts in health education and health promotion, one cardiologist, and one nursing and expert in instrumentation) was asked to evaluate the questionnaire. The content validity ratio (CVR) and content validity index (CVI) was used to confirm the quantitative content validity.

The CVR was determined by dividing the questionnaire's design score into three categories: “the item is necessary, the item is useful, but “review,” it is not necessary, there is no need.” The board of experts, which was composed of 10 experts, used this score to calculate the CVI. Using the Lawshe table index, each item with the item impact score above 1.5 and CVR above 0.79, and the questions pertaining to that item were deemed essential and significant, thus being retained for more examination [26]. Based on Cronbach's alpha, the overall reliability was calculated to be 0.89. Also, the reliability of knowledge, attitude, self-efficacy, reinforcing factors, and enabling factors was calculated to be 0.79, 0.86, 0.84, 0.79, and 0.81, respectively.

### 2.8. LSQ

The LSQ of Lali et al., the validity of which was confirmed [25], was used to assess lifestyle. The questionnaire included:

1. The seven questions physical exercise (e.g., “I exercise at least several times per week”) and health (e.g., “I try to keep my body healthy and fit”).On a four-point Likert scale, responses ranged from never = 1, sometimes = 2, frequently = 3, and always = 4.
2. The seven questions about weight control (e.g., “I always keep my weight at an optimal level”) and nutrition (e.g., “I consume fruits and vegetables at least five times per day”). On a four-point Likert scale, responses ranged from never = 1, sometimes = 2, frequently = 3, and always = 4.
3. The seven questions about mental health (e.g., “I can control stress”). On a four-point Likert scale, responses ranged from never = 1, sometimes = 2, frequently = 3, and always = 4.
4. The seven questions about spiritual health (e.g., “I try to do worthwhile things”). On a four-point Likert scale, responses ranged from never = 1, sometimes = 2, frequently = 3, and always = 4.

The reliability of this tool was confirmed in previous studies [27].

### 2.9. Intervention

**Figure 1:**
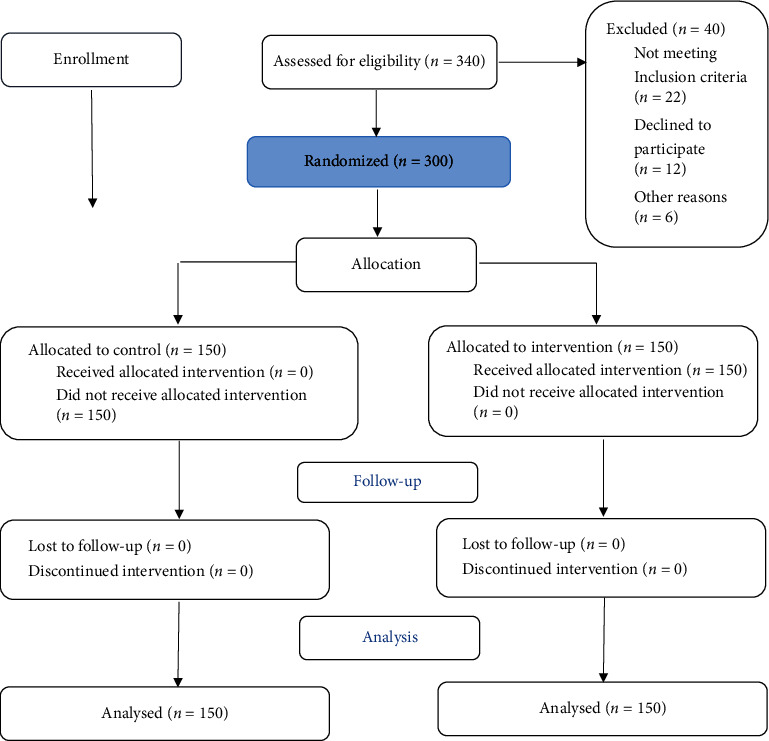
Flowchart of the study.

In this study, there were two groups: a control group that received no intervention and an intervention group that received the training program based on the PRECEDE model's structures.  Figure 1 illustrates the study's flowchart. Before the intervention, the purpose of the study was explained to the participants. Prior to the intervention, the questionnaires were completed by both groups. At the outset of the procedure, the systolic and DBPs were measured. Then, based on the results of the pretest, ten 45 to 50 min sessions At the Fasa Health Center, the sessions were led by one Ph.D. in health education and health promotion, one cardiologist, and one Ph.D. in nursing, with the assistance of two experts in noncommunicable diseases that were consisting of lectures, Q and A, group discussion, practical demonstration, video excerpts, and PowerPoint were conducted.

The sessions included the importance of proper lifestyle, the definition of Hypertension and its complications, hypertension control, weight control and nutrition, physical exercise, mental health, and stress control. At least five educational and motivational messages were sent daily to the WhatsApp group and a WhatsApp group was formed, and an educational booklet was given to the patients. In one of the sessions, attended by a family member, Health Center staff, and the doctor, the role of subjective norms, social support, and creating continuous motivation for the continuation or repetitive behavior was emphasized (Table 1). 6 months after the intervention, the data were gathered using the same questionnaire in two groups. Finally, the data were analyzed and compared.

### 2.10. Statistical Analysis

Data were analyzed using the SPSS version 22 software. The normality of the data was first measured using the Kolmogorov–Smirnov test. Comparison of the mean scores of lifestyle dimension and hypertension in the two groups before and after the intervention was done using pair *t*-test. The multilevel modeling and comparison of demographic information of the participants in the two groups were done using independent *t*-test. A *p* value of < 0.05 was considered significant.

## 3. Results

This study was performed on 300 Hypertensive Patients. The normality of the data was measured through the Kolmogorov–Smirnov test. The mean age of the intervention and control groups were 51.13 ± 11.58 and 52.57 ± 11.73 years, respectively (*p*=0.362). As shown in Table 2, there is no significant difference between the two groups regarding demographic characteristics, including age, gender, and education (Table 2).

The results showed that, the experimental group had a significant increase 6 months after the intervention in knowledge, attitude, self-efficacy, enabling factors, and reinforcing factors (*p* < 0.001) (Table 3).

The results showed that the experimental group had a significant increase 6 months after the intervention in terms of physical exercise and health variables, nutrition and weight control, mental health, and spiritual health (*p* < 0.001) (Table 4)

Also, the mean blood pressure [both diastolic (−6.27) and systolic (−10.14)] in the experimental group was significantly reduced after the intervention (*p* < 0.001) (Table 5).

## 4. Discussion

This research aimed to determine the effect of educational intervention by application of PRECEDE-PROCEED model on lifestyle change in hypertensive patients in the villages of Fasa City, Fars Province, Iran.

The study results showed that the intervention based on the PRECEDE-PROCEED model effectively changed patients' lifestyles. This confirms the effectiveness of the model-based educational intervention on patients' lifestyles. The result of Moshki et al. and Wahyuningsih et al. studies were consistent with the results of this study [28, 29]. Also, the mean knowledge score significantly increased in the experimental group 6 months after the educational intervention. Education through lectures, group discussions, Q and A, forming a WhatsApp group to exchange information and sending educational and motivational messages, and providing the experimental group with educational booklets increased their knowledge on healthy lifestyle and hypertension. In studies by Moshki et al. and Mosavi et al., educational intervention increased participants' knowledge [27, 30].

After the educational intervention, a significant increase was observed in the experimental group regarding the mean score of attitude, indicating the effectiveness of the PRECEDE-PROCEED model. In this study, intervention through group discussions and Q and A significantly improved the experimental group's attitude toward a healthy lifestyle. The results of other study conducted by Dizaji et al., was consistent with this study [31].

The results of this study indicated an increase in the mean self-efficacy score in the experimental group 6 months after the educational intervention. The present study used many strategies to improve self-efficacy including encouraging patients to proper diet, physical exercise, stress control, and mental health promotion and in five and sex sessions for increase self-efficacy, the researcher played the role of a facilitator and in order to influence the automaticity of people, Solutions including simplifying preventive behaviors and expressing successful experiences by using the opinion and experience of other people in the group and how they overcome the problems of implementing treatment protocols were emphasized and it was stated that you can!. Also, in similar study by Said et al, educational intervention increased self-efficacy [32].

The results of this study indicated a significant difference between the two groups in terms of enabling factors 6 months after the educational intervention. Providing educational content through booklets, video clips, Q and A; access to healthcare staff, follow-up activity, and forming a WhatsApp group to exchange information increased the ability of patients to change their lifestyle and control hypertension positively. The present study's results were consistent with other studies conducted in Iran [33, 34].

The results of the present study showed a significant difference between the experimental and control groups regarding the reinforcing factors 6 months after the educational intervention, indicating the effectiveness of the PRECEDE-PROCEED model. Holding a session for family members, physicians, and staff of health centers as subjective norms and social supports, as well as providing educational content through group discussions, Q and A, and forming a WhatsApp group increased the mean score of the reinforcing factors in the experimental group. The results of other studies such as Ahmed Osman Mohamed et al. and Barasheh et al., were consistent with this study [35, 36].

In the present study, the experimental group's mean scores of the PRECEDE-PROCEED model constructs and lifestyle dimensions (physical exercise, weight control, and nutrition, mental, and spiritual health) significantly increased 6 months after the educational intervention. The results of this study were consistent with the results of other studies conducted by Babaei-Sis et al. and Shayesteh et al. [20, 37].

Also, the experimental group's mean systolic and DBP significantly decreased after the intervention. In this study, holding educational sessions, providing booklets, correcting nutrition and physical activities, improving mental and spiritual health, and involving social supports such as family members, physicians, and health center staff significantly improved patients' lifestyles and blood pressure. The results of this study were consistent with the results of other studies conducted by Prasanna et al., Estrada et al., and Khani Jeihooni et al. [38–40].

### 4.1. Strengths and Limitations

The study was conducted on rural patients with hypertension who have less access to treatment facilities. Another strength of this study was to involve health center staff and the model-based educational intervention, which provides researchers with an academic, practical, and useful guide and framework for needs assessment, intervention design, intervention implementation and different techniques were used in educational sessions.

Limitations of the present study include a low sample size. Asking for free participation may be one reason for the small number of samples in the present study. The use of self-reporting tools is another limitation of the present study. Another limitation of the present study is the study environment that cultural, social, or organizational factors of this environment could have affected the results of this study.

## 5. Conclusion

The results of this study showed the effectiveness of lifestyle education based on the PRECEDE-PROCEED model in controlling hypertension. It also highlights the need to pay further attention to the education aimed at controlling hypertension through a healthy lifestyle and correct behavioral habits. Education, improving patients' performance, and preventing the disease could form correct behavioral patterns, leading to a healthy lifestyle. Due to the vulnerability of hypertensive patients and their insufficient knowledge of the disease, measures such as changing their attitude, increasing family knowledge, and providing educational programs through mass media may play an important role in changing patients' lifestyles. According to the PRECEDE-PROCEED model, special attention should be paid to reinforcing factors such as family, friends, and health center staff.

## Figures and Tables

**Table 1:** Educational interventions in the intervention group.

Session number	The structures of this model for content	Time of perform	Duration of this program	Session description
The first and second session	Knowledge	The first and second week	45–50 min	The sessions included the importance of proper lifestyle, definition of hypertension and its complications, hypertension control, weight control and nutrition, physical exercise, mental health, and stress control
The third session	Attitude	The third week	45–50 min	In this part of the training, the seriousness of the complications and injuries caused by the disease was stated, and in this regard, at the same time, pictures of the actual condition of patients in special hospital wards were presented with a slide show. Describe the complications of the disease. During the intervention, an effort was made to make the participants actively participate in the educational program. Also, in order to bring closer their attitude to the depth of the danger and the seriousness of the complications, a report was presented on the high number of deaths due to this disease in the city
The five and six session	Self-efficacy	The five and 6 week	45–50 min	The participants were asked to state and compare the obstacles and benefits of preventive behavior and to state the problems and necessary solutions. At this time, the researcher played the role of a facilitator and in order to influence the automaticity of people, solutions including simplifying preventive behaviors and expressing successful experiences by using the opinion and experience of other people in the group and how they overcome the problems of implementing treatment protocols were emphasized and it was stated that you can!
The seven and eight session	Enabling factors	The seven and 8 week	45–50 min	In these sessions provided, information needs fit into participants' lifestyle/schedule/living conditions
The nine and ten session	Reinforcing factors	The nine and 10 week	45–50 min	These sessions are performed by a family member, health center staff, and the doctor to participate in communication with healthcare providers and social networking (they provide social support and create continuous motivation for the continuation or repetitive behavior)
Total	All structures of this model for content	2.5 months	450–500 min	—

**Table 2:** Demographic information of studied patients.

Variables		Experimental group		Control group		*p* value
		Number	Percentage	Number	Percentage	
Education	Illiterate	8	5.33	4	2.66	< 0.159
	Primary school	16	10.67	22	14.67	
	Secondary school	42	28	38	25.33	
	High school	68	45.33	64	42.67	
	University	16	10.67	22	14.67	

Gender	Female	86	57.33	94	62.67	< 0.092
	Male	64	42.67	56	37.33	

Marital status	Single	7	4.67	9	6	< 0.146
	Married	138	92	132	88	
	Divorced	3	2	4	6	
	Widowed	2	1.33	3	2	

Age	30–39	35	23.33	30	20	< 0.104
	40–49	30	45	48	32	
	50–59	38	25.33	34	22.67	
	≥ 60	32	21.34	38	25.33	

*Note*: The independent t-test (*p* < 0.05).

**Table 3:** Comparison of mean score of cues model in the two groups before and 6 months after the intervention.

Variable	Group	Before intervention M ± SD	6 months after intervention M ± SD	Mean difference	*p* value
Knowledge	Experimental	7.25 ± 3.40	20.18 ± 3.32	+12.93	< 0.001⁣^∗^
	Control	7.87 ± 3.18	8.09 ± 3.14	+0.22	< 0.202
	*p* value	< 0.188	< 0.001⁣^∗∗^		

Attitude	Experimental	28.44 ± 8.77	76.72 ± 4.36	+48.28	< 0.001⁣^∗^
	Control	26.30 ± 7.14	29.64 ± 7.08	+3.34	< 0.195
	*p* value	< 0.133	< 0.001⁣^∗∗^		

Self-efficacy	Experimental	20.25 ± 5.14	50.12 ± 5.31	+29.87	< 0.001⁣^∗^
	Control	22.16 ± 5.06	24.02 ± 5.66	+1.86	< 0.210
	*p* value	< 0.182	< 0.001⁣^∗∗^		

Enabling factors	Experimental	18.34 ± 5.21	50.55 ± 5.09	+32.21	< 0.001⁣^∗^
	Control	17.36 ± 5.11	19.13 ± 5.20	+1.77	< 0.233
	*p* value	< 0.273	< 0.001⁣^∗∗^		

Reinforcing factors	Experimental	8.22 ± 2.14	20.14 ± 2.10	+11.92	< 0.001⁣^∗^
	Control	9.04 ± 2.47	10.08 ± 2.35	+1.04	< 0.290
	*p* value	< 0.207	< 0.001⁣^∗∗^		

Abbreviations: M, mean; SD, standard deviation.

⁣^∗^Paired t-test (*p* <  0.05), ⁣^∗∗^Multilevel modeling (*p* <  0.05).

**Table 4:** Comparison of mean score of lifestyle dimensions in the experimental and control groups.

Variable	Group	Before intervention M ± SD	6 months after intervention M ± SD	Mean difference	*p* value
Physical exercise	Experimental	12.18 ± 2.52	23.42 ± 2.34	+11.24	< 0.001⁣^∗^
	Control	13.03 ± 2.60	13.63 ± 2.64	+0.60	< 0.284
	*p* value	< 0.372	< 0.001⁣^∗∗^		

Weight control and nutrition	Experimental	14.03 ± 2.16	22.90 ± 2.66	+8.87	< 0.001
	Control	13.47 ± 2.09	14.14 ± 2.15	+0.67	< 0.226
	*p* value	< 0.318	< 0.001⁣^∗∗^		

Mental health	Experimental	12.88 ± 2.62	23.38 ± 2.66	+10.5	< 0.001⁣^∗^
	Control	13.28 ± 2.33	14.55 ± 2.30	+1.27	< 0.177
	*p* value	< 0.289	< 0.001⁣^∗∗^		

Spiritual health	Experimental	15.07 ± 2.23	23.46 ± 2.41	+8.39	< 0.001⁣^∗^
	Control	13.60 ± 2.04	14.45 ± 2.58	+0.85	< 0.172
	*p* value	< 0.269	< 0.001⁣^∗∗^		

Abbreviations: M, mean; SD, standard deviation.

⁣^∗^Paired t-test (*p* <  0.05), ⁣^∗∗^Multilevel modeling (*p* <  0.05).

**Table 5:** Comparison of the mean score of hypertension in the two groups before and 6 months after the intervention.

Variable	Group	Before intervention M ± SD	6 months after intervention M ± SD	Mean difference	*p* value
Systolic hypertension	Experimental	131.28 ± 15.65	121.14 ± 14.60	−10.14	< 0.001⁣^∗^
	Control	132.51 ± 15.88	133.01 ± 15.83	−0.5	< 0.154
	*p* value	0.163	0.001⁣^∗∗^		

Diastolic hypertension	Experimental	80.65 ± 9.40	74.38 ± 7.58	−6.27	< 0.001⁣^∗^
	Control	82.31 ± 9.25	82.52 ± 9.29	−0.21	< 0.355
	*p* value	< 0.074	< 0.001⁣^∗∗^		

Abbreviations: M, mean; SD, standard deviation.

⁣^∗^Paired *t*-test (*p* <  0.05), ⁣^∗∗^Multilevel modeling (*p* <  0.05).

## Data Availability

The datasets used and/or analyzed during the current study are available from the corresponding author upon reasonable request.

## Endnotes

^1^Fruits and vegetables (FV).
